# Comparative Analysis of HPV Detection Efficiency: Evaluating Cobas 8800 Performance in Vaginal Self-Sampling versus Clinician-Collected Samples at a Regional Thai Hospital

**DOI:** 10.3390/diagnostics14192177

**Published:** 2024-09-29

**Authors:** Umaporn Ruttanamora, Pinsawitar Thongsalak, Araya Sammor, Sirinart Chomean, Chollanot Kaset

**Affiliations:** 1 Graduate Program in Medical Technology, Faculty of Allied Health Sciences, Thammasat University, Pathum Thani 12120, Thailand; umaporn.rut@allied.tu.ac.th; 2 Department of Medical Technology, Faculty of Allied Health Sciences, Thammasat University, Pathum Thani 12120, Thailand; pinsawitar.tho@dome.tu.ac.th (P.T.); sirinat.c@allied.tu.ac.th (S.C.); 3 Faculty of Medicine, Thammasat University, Pathum Thani 12120, Thailand; orohyeah@gmail.com; 4 Thammasat University Research Unit in Medical Technology and Precision Medicine Innovation, Thammasat University, Pathum Thani 12120, Thailand

**Keywords:** hrHPV, Ct value, Cobas 8800, self-sampling, clinician collected

## Abstract

Background: This study, conducted at a regional Thai hospital, assesses the comparative efficacy of self-collected versus clinician-collected samples for HPV detection using the Cobas 8800 system among Thai women aged 30–60. Methods: Our methodology involved analyzing 1541 self-collected and 1398 clinician-collected samples. Results: The results show a statistically significant mean difference in cycle threshold (Ct) values favoring clinician-collected samples (1.53; 95% CI: 1.18–1.87, *p* < 0.0001). This pattern was consistent across various age groups, with the most pronounced differences noted in the oldest cohort (50–59 years), suggesting higher detection efficiency in clinician-collected samples. The study further explored the correlation of Ct values with cytological and histological outcomes, where clinician-collected samples demonstrated superior diagnostic performance, particularly in identifying LSIL and HSIL conditions, evidenced by AUC values of 0.793 and 0.866, respectively. While self-sampling remains a viable method, with sensitivity reaching up to 48.84% for LSIL and 46.15% for HSIL, clinician collection proved more accurate, likely influencing future national screening policies. Conclusions: This work underscores the need for robust sample collection methods and the importance of ongoing enhancements to self-sampling assays and techniques to ensure their efficacy in cervical cancer screening programs.

## 1. Introduction

Human papillomavirus (HPV) remains a significant global health concern, directly linked to cervical cancer incidences. In 2020 alone, there were an estimated 604,127 new cases and 341,831 deaths attributed to cervical cancer worldwide [1,2]. Thailand continues to grapple with a high burden of cervical cancer, reporting approximately 6000 new cases annually [1]. This incidence significantly exceeds the WHO’s target for elimination, highlighting a critical need for enhanced screening measures [3].

The WHO recommends HPV DNA testing as the primary method for cervical cancer screening, starting at age 30 and repeating every five to ten years [4]. This method is preferred for its superior efficacy over traditional screening approaches such as visual inspection with acetic acid (VIA) or cytology [5]. Despite setting a target to conduct molecular HPV DNA testing on 1,828,298 individuals in fiscal year 2023, only 829,096 (45.9%) were actually screened [6]. Of these, 15,953 (1.9%) tested positive for high-risk HPV types 16/18 and an additional 55,604 (6.6%) for other types of HPV [6]. Those testing positive for non-16/18 types were further evaluated with liquid-based cytology (LBC), resulting in 9071 positive cases (25.5%) that necessitated further investigation through colposcopy [6].

The adoption of self-sampling has marked a significant advancement in expanding access to cervical cancer screening. This technique not only broadens screening coverage but also mitigates accessibility issues, particularly among women who are hesitant to participate in traditional screening programs due to barriers like fear, embarrassment, or logistical challenges [7]. Studies from Thailand and various international settings confirm that self-collected samples for high-risk HPV (hrHPV) testing align closely with clinician-collected samples, providing a sensitive and specific alternative that has gained acceptance among women [1,2,8,9,10].

During the COVID-19 pandemic, self-sampling demonstrated its value by enabling continuous screening while maintaining social distancing protocols [1]. This adaptability highlights the method’s potential contribution toward meeting the WHO’s objective of eradicating cervical cancer as a public health threat by 2030 [2,4].

Our investigation at Chonburi Hospital aims to delve deeply into these findings by assessing the comparative efficacy of self-sampling against clinician-collected samples in routine HPV screening with the Cobas 8800 system, targeting Thai women aged 30–60. This study is poised to offer crucial insights into the viability and precision of self-sampling within the Thai context, potentially influencing future strategies for cervical cancer screening and shaping public health policy.

## 2. Materials and Methods

### 2.1. Cervical Cancer Screening Guidelines in Thailand

General Information about Cervical Sample Collection in Thailand: Chonburi Hospital participates in a project by the National Cancer Institute, Department of Medical Services, Ministry of Public Health, Thailand, that offers cervical cancer screening for women aged 30–60 using the HPV test method. This initiative utilizes the Cobas 8800 system, employing real-time PCR to detect genetic material from 14 high-risk HPV strains, ensuring comprehensive and accurate results.

**Figure 1:**
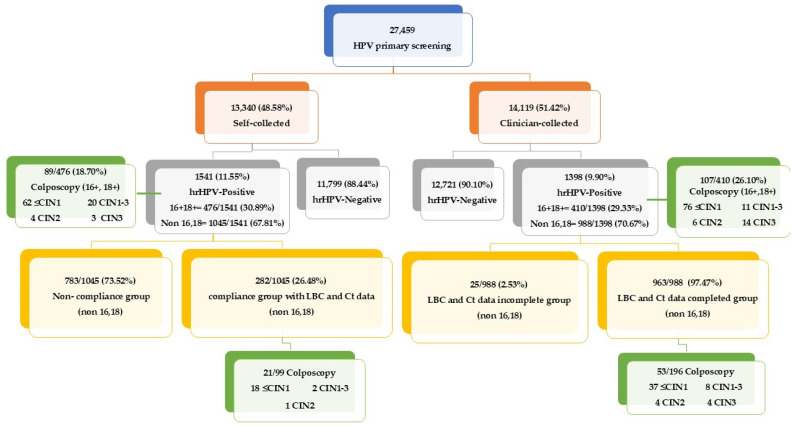
Flowchart of the cervical cancer screening guidelines in Thailand, depicting the primary HPV screening process and subsequent diagnostic pathways. The chart outlines the initial HPV primary screening and the distribution of self-collected versus clinician-collected samples. Positive hrHPV cases are categorized by HPV types 16, 18, and other high-risk types, with follow-up actions including colposcopy examinations based on HPV-type positivity. The subsequent findings from the colposcopy, classified according to the 2014 Bethesda System, are shown for each category. Adequacy of baseline cytology is assessed with corresponding follow-up outcomes for non-16,18 HPV types.

Pathway and Subsequent Management for Vaginal Self-Samples: Self-collected vaginal sampling offers greater accessibility and privacy. Women receive self-sampling kits distributed by the Contracting Unit for Primary Care (CUP) to subdistrict health-promoting hospitals or primary care units. After collection, these samples are sent to Chonburi Hospital for analysis. If a self-collected sample tests positive for HPV types other than 16/18, reflex liquid-based cytology (LBC) is performed in the same vial. Abnormal LBC findings lead to further evaluation via colposcopy to check for cervical pathology (Figure 1).

- New Sample Collection Requirement: If an HPV self-sampling test is deemed inadequate due to insufficient cellular material or poor sample quality, our protocol requires that a new self-collected sample be obtained. This sample is then sent back to the laboratory for further HPV DNA testing.
- Follow-up for Positive Non-16/18 Cases: In the event of a non-16/18 positive result, participants are requested to follow up with a clinician-obtained sample. This subdivides into a compliance group with complete LBC and Ct data and a non-compliance group with participants who did not follow up as required.
- Management of HPV Types 16 or 18: Participants with samples positive for HPV types 16 or 18, whether self-collected or clinician-collected, must undergo colposcopy to further assess and manage potential high-risk lesions.

Pathway and Subsequent Management for Clinician-Obtained Cervical Samples: Clinician-collected sampling is the second method utilized, with samples sent through the CUP to Chonburi Hospital for HPV DNA testing. For samples testing positive for HPV types 16/18, they are directly referred for colposcopy confirmation, bypassing initial cytology to ensure prompt attention to potential high-risk cases. For non-16/18 types (Figure 1):

- LBC and Ct Data Completed Group: Samples from this group are used directly from the HPV DNA test step for LBC without the need for a new collection.
- LBC and Ct Data Incomplete Group: A small subset where either LBC or Ct data, or both, are missing.

Colposcopy and Further Assessment: If LBC results from clinician-collected samples reveal abnormalities at or above the threshold of ASCUS, a colposcopy examination is recommended using the 2014 Bethesda System. Ambiguities in disease severity are classified as CIN1–3*, necessitating further diagnostic clarity, which may involve additional colposcopy or biopsy.

### 2.2. Self-Collection and Clinician Collection

Samples collected by clinicians are sent from the Department of Obstetrics and Gynecology, Chonburi Hospital, CUP, sub-district health-promoting hospital, or the primary care unit of the sub-district health-promoting hospital. Samples are collected by clinicians using a cervical brush with a broom-like device (Hologic, Marlborough, MA, USA) and then placed into ThinPrep PreservCyt Solution (Preservative solution) with a volume of no less than 20 mL.

On the other hand, samples collected from the cervix by self-sampling are obtained by women either at home, at a contracting unit for primary care (CUP), a sub-district health-promoting hospital, or the primary care unit of a sub-district health-promoting hospital. A FLOQ Swab (Copan Diagnostics Inc., Murrieta, CA, USA) was used to collect cervical cells from the samples collected from the cervix by self-sampling, and then the samples were immersed in Roche Cell Collection Medium (RCCM) (Roche Molecular Systems, Inc., South Branchburg, NJ, USA) with a volume of no less than 20 milliliters. In the case of detecting non-16/18 HPV, women must provide a new sample for LBC testing by a clinician. Chonburi Hospital provides cervical cancer screening using HPV DNA testing with the Cobas 8800 system, utilizing real-time PCR to detect high-risk HPV genetic material from all 14 strains. Internal quality control systems are in place for each sample, including the beta-globin gene, to ensure sample and internal quality control.

### 2.3. Data Collection

This research is a retrospective cross-sectional study comparing Ct value between self-collected and clinician-collected samples in routine HPV screening for the detection of positive cytology and CIN2 or CIN3 histology. The study compares self-collected samples with clinician-collected samples using the Cobas 8800 system among Thai women at Chonburi Hospital. It utilizes data from HPV DNA tests in Thai women aged 30–60 from 24 May 2021 to 6 July 2023 under the National Cancer Institute’s HPV screening program. The sample size includes 13,340 self-collected and 14,119 clinician-collected specimens, totaling 27,459 participants.

The inclusion criteria for the sample selection in this study are as follows: The study involves Thai women aged 30–60 years who have not undergone screening in the past five years. It includes data from samples sent for cervical cancer screening using the HPV DNA test during the study period. The data are only from samples collected both by clinicians and through self-collection. The samples collected are cervical swabs stored in PreservCyt solution or Roche Cell Collection Medium (RCCM). The exclusion criteria for the study are as follows: Samples from HPV DNA tests that lack either the HPV DNA test results or LBC results are excluded. Also excluded are samples without confirmatory colposcopy results from healthcare units; samples with inappropriate volume or quality, such as those stored for over three months; and samples exhibiting hemolysis.

Data regarding cytology test results using the LBC method were obtained from the Pathology Clinic of Chonburi Hospital through the Hospital Information System (HIS) of Chonburi Hospital, the HPV connect program by Roche Diagnostics (Bangkok, Thailand) Company, and the HPVcxs2020 screening program. These systems are interconnected to facilitate data retrieval for screening history, sending screening data, and confirming colposcopy results. The collected information is also stored on the website of the National Cancer Institute at www.nci.go.th (accessed on 1 April 2024).

### 2.4. Statistical Analyses

Among women with a positive result for high-risk human papillomavirus (hrHPV), we calculated the mean difference in Ct values between samples collected by the women themselves and those collected by clinicians. This calculation was stratified by age category, cytology, and histology. The Shapiro–Wilk test was employed to assess the distribution of the data. Subsequently, either the *t*-test or Mann–Whitney U test was utilized to examine differences in mean or mean rank of Ct values, respectively. This analysis was conducted based on age groups (30–39, 40–49, and 50–59 years), types of HPV DNA (16+, 18+, or non 16,18+), severity of cytology (negative, LSIL, and HSIL), and histology (≤CIN1, CIN2, and CIN3). Regarding the diagnostic performance of Ct values in distinguishing cytology results as LSIL and HSIL, as well as distinguishing histology results as CIN2 and CIN3, we employed ROC curve analysis. This analysis displayed the area under the curve (AUC), sensitivity, and specificity. Statistical analyses were performed using MedCalc^®^ Statistical Software version 22.021 (MedCalc Software Ltd., Ostend, Belgium).

## 3. Results

### 3.1. Analysis of Ct Values for HPV Detection across Various Age Groups

In a comprehensive evaluation at Chonburi Hospital, the study analyzed the mean Ct values for HPV detection across various age segments using self-collected and clinician-collected samples with the Cobas 8800 system. A total of 1541 self-collected and 1398 clinician-collected samples were compared, with a statistically significant mean difference favoring clinician-collected samples (1.53; 95% CI: 1.18–1.87, *p* < 0.0001). When stratified by age, the differences between collection methods were evident across all groups. Among the youngest cohort (30–39 years), which constituted 55.75% of the self-collected group but only 41.35% of the clinician-collected group, the mean Ct value was 29.15 ± 4.43 for self-collection versus 27.48 ± 4.81 for clinician collection, with a mean difference of 1.67 (95% CI: 1.18–2.15, *p* < 0.0001). The middle age group (40–49 years) represented 34.00% of self-collected and 37.05% of clinician-collected samples, with mean Ct values of 29.09 ± 4.66 and 27.71 ± 5.03, respectively, and a mean difference of 1.38 (95% CI: 0.79–1.96, *p* < 0.0001). Finally, the oldest group (50–59 years), accounting for 10.25% of the self-collected and 21.60% of the clinician-collected samples, showed a mean Ct value of 30.00 ± 4.67 in self-collection against 28.06 ± 5.10 in clinician collection, with the mean difference being the highest at 1.94 (95% CI: 0.99–2.90, *p* < 0.0001). These results underscore the trend of lower Ct values in clinician-collected samples across all age groups, with the disparity being more pronounced in the oldest age bracket. The proportional representation within each age group suggests varying acceptability or accessibility to self-sampling, which may influence future public health strategies for cervical cancer screening in Thailand.

### 3.2. Analysis of Ct Values for HPV Detection across Various hrHPV Types

**Figure 2:**
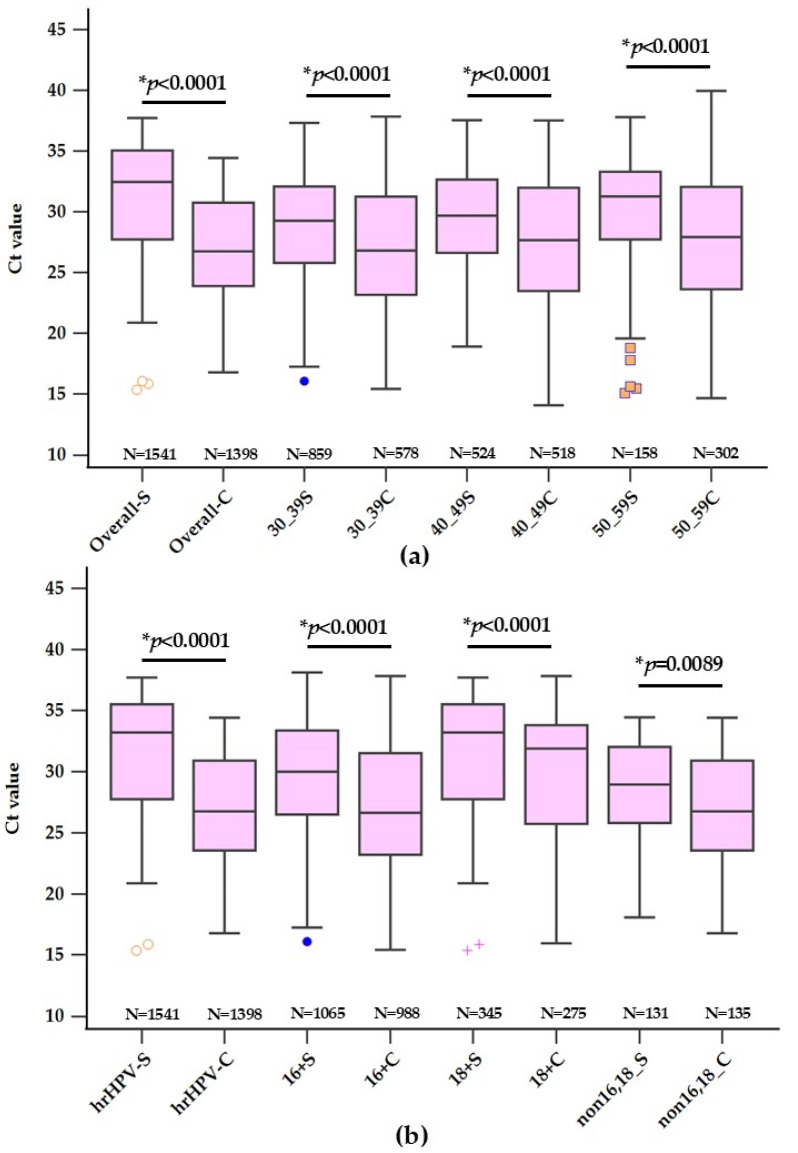
Boxplot comparison of Ct values by age group (**a**) and HPV types (**b**) in self-collected and clinician-collected samples. Note: An asterisk (*) indicates statistically significant differences in Ct values (*p* < 0.0001). Outliers are represented by colored dots outside the box plots.

A comparative study was conducted analyzing the mean Ct values associated with different types of high-risk HPV (hrHPV), specifically types 16,18, and non 16,18, in self-collected versus clinician-collected samples. Across the total of 1541 self-collected and 1398 clinician-collected samples, the overall mean Ct values were 29.22 ± 4.54 and 27.69 ± 4.96, respectively, with a significant mean difference of 1.53 (95% CI: 1.18–1.87, *p* < 0.0001). Stratification by hrHPV type revealed that for HPV 16, which comprised 22.39% of self-collected and 19.67% of clinician-collected samples, the mean Ct value was 30.36 ± 4.47 for self-collected samples, notably higher than the clinician-collected samples’ mean of 28.13 ± 5.25, with a mean difference of 2.23 (95% CI: 1.46–3.00, *p* < 0.0001). HPV 18 was identified in 8.50% of self-collected and 9.66% of clinician-collected samples, exhibiting a mean Ct value of 31.58 ± 4.93 for self-collected and 29.93 ± 5.24 for clinician-collected samples, with a mean difference of 1.65 (95% CI: 0.41–2.88, *p* = 0.0089). The non 16,18 types, representing the majority, with 69.11% of self-collected and 70.67% of clinician-collected specimens, showed mean Ct values of 28.56 ± 4.33 and 27.26 ± 4.75, respectively, and a mean difference of 1.30 (95% CI: 0.90–1.69, *p* < 0.0001) (Figure 2).

These data not only underscore the consistent pattern of lower Ct values in clinician-collected samples across all HPV types but also reflect the distribution of hrHPV types in the population studied. The substantial proportion of non 16,18 types indicates a prevalent risk that could be pivotal in shaping targeted screening and vaccination strategies within the Thai healthcare framework.

### 3.3. Comparative Analysis of HPV Detection and Cytological Outcomes in Self-Collected vs. Clinician-Collected Samples

**Figure 3:**
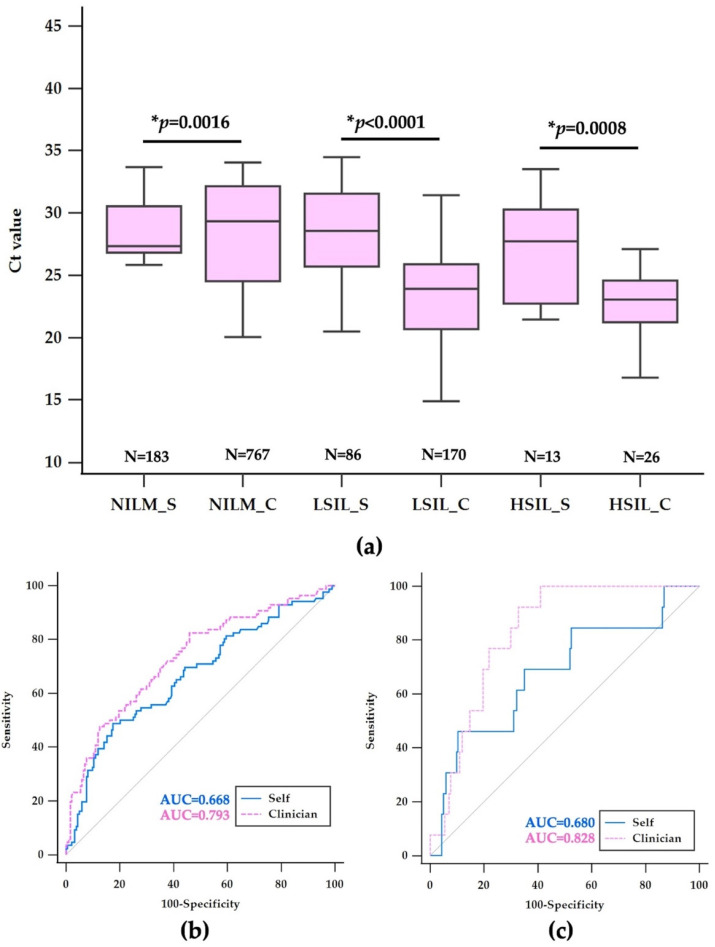
Boxplot comparison of Ct values by cytology grading results (**a**) and ROC curves for Ct value distribution and diagnostic accuracy in the detection of LSIL (**b**) and HSIL (**c**) in self-collected versus clinician-collected samples. Note: An asterisk (*) indicates statistically significant differences in Ct values (*p* < 0.0001). The grey dotted line represents the line of no discrimination, indicating performance equivalent to chance.

In this detailed analysis at Chonburi Hospital, patients who tested positive for non-16,18 hrHPV types and underwent LBC were assessed for cytological outcomes. The study encompassed 282 self-collected and 963 clinician-collected samples, all of which were adequate for reflex cytology in the same sample. Among the self-collected specimens, 183 (64.89%) were cytologically negative, with a mean Ct value of 29.44 ± 3.78, compared to 767 (79.65%) of clinician-collected samples, with a mean Ct value of 28.37 ± 4.17, yielding a statistically significant mean difference of 1.07 (95% CI: 0.41–1.73, *p* = 0.0016), as shown in Table 1 and Figure 3a. The positive cytology group was further divided into LSIL and HSIL categories. Within the self-collected samples, LSIL was diagnosed in 86 (30.49%) cases, with a mean Ct value of 26.88 ± 4.56, substantially higher than that in the clinician-collected LSIL group, which constituted 170 (17.65%) cases, with a mean Ct value of 23.02 ± 4.66. This difference was pronounced, with a mean difference of 3.86 (95% CI: 2.41–4.88, *p* < 0.0001). For HSIL, 13 (4.62%) cases were identified in self-collected samples, with a mean Ct value of 26.80 ± 4.29, whereas clinician-collected HSIL cases accounted for 26 (2.70%), with a mean Ct value of 22.39 ± 3.13, indicating a significant mean difference of 4.41 (95% CI: 1.96–6.85, *p* < 0.0008), as shown in Table 1 and Figure 3a.

Overall, the percentage of negative results was higher in clinician-collected samples, while self-collected samples had a higher percentage of positive results when broken down into LSIL and HSIL. These findings suggest that while self-collected samples yielded a higher Ct value, indicating a lower viral load detection, they also showed a higher proportion of abnormal cytological findings, highlighting the importance of considering sample collection methods in cervical cancer screening and subsequent diagnostic procedures such as colposcopy.

In this study, out of 1045 self-collected samples from individuals who tested positive for non 16,18 high-risk HPV types, only 282 (26.48%) had adequate baseline cytology with a reportable Ct value. This suggests that a significant proportion of self-collected samples may not have met the cytological criteria necessary for a valid analysis, which could influence the number of confirmed cases and subsequent colposcopy referrals. Conversely, the clinician-collected samples exhibited a higher rate of adequacy, with 963 out of 988 samples (97.47%) achieving adequate baseline cytology with a reportable Ct value for the same non-16,18 HPV types. The higher adequacy rate indicates that clinician-collected samples are more likely to be sufficient for cytological evaluation, thus leading to more reliable screening outcomes and potentially more accurate indications for further diagnostic procedures such as colposcopy.

The data provided reflect the performance analysis of Ct values in detecting high-risk HPV in LSIL and HSIL through self-collected and clinician-collected samples. The performance is measured in terms of sensitivity, specificity, and area under the receiver operating characteristic curve (AUC). For the detection of LSIL, self-collected samples with a Ct cut-off value of ≤26.07 showed a sensitivity of 48.84% (95% CI: 37.9–59.9) and a specificity of 82.51% (95% CI: 76.2–87.7), resulting in an AUC of 0.668. Clinician-collected samples with a Ct cut-off value of ≤24.95, however, exhibited a higher sensitivity of 69.41% (95% CI: 61.9–76.2) and a slightly lower specificity of 77.84% (95% CI: 74.7–80.7), with an improved AUC of 0.793, indicating a better overall performance in LSIL detection, as shown in Figure 3b and Table 2. In the context of HSIL detection, self-collected samples with a Ct cut-off of ≤24.46 had a sensitivity of 46.15% (95% CI: 19.2–74.9) and a specificity of 86.63% (95% CI: 84.3–93.6), with an AUC of 0.680. Clinician-collected samples, with a higher Ct cut-off value of ≤25.47, demonstrated a substantially greater sensitivity of 88.46% (95% CI: 69.8–97.6) and a specificity of 73.01% (95% CI: 69.7–76.1), yielding a higher AUC of 0.866. This indicates a superior diagnostic performance for HSIL, as shown in Figure 3c and Table 2.

The AUC values, a measure of the test’s overall ability to discriminate between those with and without the disease, were consistently higher in clinician-collected samples for both LSIL and HSIL. The clinician-collected samples achieved a notably higher sensitivity for both LSIL and HSIL, while maintaining a commendable specificity, especially in the case of HSIL, where the sensitivity was notably high. This detailed analysis highlights the differences in the diagnostic performance of self-collected versus clinician-collected samples for HPV testing with respect to LSIL and HSIL. It shows that clinician-collected samples tend to provide a higher diagnostic accuracy, as reflected by the higher AUC values, and should be considered in clinical settings where precise HPV detection is critical.

### 3.4. Histological Findings and Ct Value Analysis in hrHPV-Positive Samples

Table 2 provides a comparison of histological findings and corresponding mean Ct values from self-collected and clinician-collected samples among women who tested positive for high-risk HPV, including non-16,18 types as well as 16 and 18 types, following LBC at Chonburi Hospital. The histological diagnosis post-colposcopy stratifies the outcomes into less than or equal to CIN1 (≤CIN1), CIN1–3*, CIN2, and CIN3, with CIN1–3* representing cases where a specific diagnosis between CIN1 and CIN3 could not be determined and will be excluded in further analysis.

**Figure 4:**
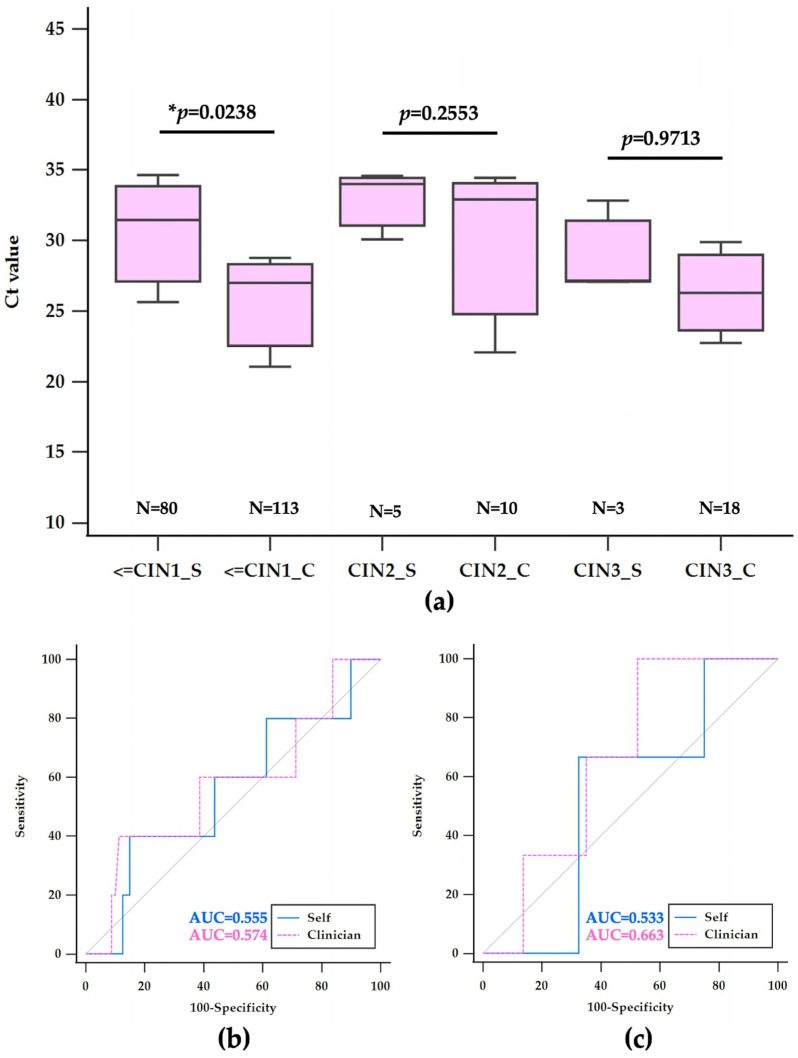
Boxplot comparison of Ct values by colposcopy grading results (**a**) and ROC curves for Ct value distribution and diagnostic accuracy in the detection of CIN2 (**b**) and CIN3 (**c**) in self-collected versus clinician-collected samples. Note: An asterisk (*) indicates statistically significant differences in Ct values (*p* < 0.0001). The grey dotted line represents the line of no discrimination, indicating performance equivalent to chance.

Out of 110 self-collected samples, 80 cases (72.72%) were diagnosed as ≤CIN1, with a mean Ct value of 29.04 ± 5.06, whereas 113 of 160 clinician-collected samples (70.64%) fell under the same category, showing a lower mean Ct value of 27.22 ± 5.76. This resulted in a statistically significant mean difference of 1.82 (95% CI: 0.24–3.40, *p* = 0.0238). For the indeterminate CIN1–3* category, 22 self-collected samples (20.00%) had a mean Ct value of 32.32 ± 4.88, significantly higher than the 19 clinician-collected samples (11.86%), with a mean Ct of 27.45 ± 5.08, yielding a mean difference of 4.87 (95% CI: 1.71–8.01, *p* = 0.0034). CIN2 was identified in 5 self-collected samples (4.55%) and 10 clinician-collected samples (6.25%), with mean Ct values of 29.75 ± 5.06 and 26.65 ± 4.61, respectively, but the mean difference of 3.1 (95% CI: 2.53–8.72) was not statistically significant (*p* = 0.2553). The least common diagnosis was CIN3, observed in 3 self-collected samples (2.73%) and 18 clinician-collected samples (11.25%), with mean Ct values closely aligned at 29.02 ± 3.30 and 28.90 ± 5.55 respectively, resulting in an insignificant mean difference of 0.12 (95% CI: 6.87–7.12, *p* = 0.9713), as shown in Figure 4a.

These findings suggest that clinician-collected samples generally yield lower Ct values, which may indicate higher viral load detection across various histological severities. The distribution of cases, particularly the higher percentage of ambiguous CIN1–3* diagnoses in self-collected samples, underscores the need for careful interpretation of self-sampling in clinical diagnostics and may influence subsequent patient management decisions.

In assessing the risk of CIN2, self-collected samples with a Ct cut-off value of greater than 33.68 demonstrated a sensitivity of 40% (95% CI: 5.3–85.3) and a specificity of 85.00% (95% CI: 75.3–92.0), with an area under the curve (AUC) of 0.555. Clinician-collected samples for CIN2, with a lower Ct cut-off value of 29.65 or less, exhibited an 80% sensitivity (95% CI: 44.4–97.5) but a notably lower specificity of 38.05% (95% CI: 29.1–47.7), resulting in an AUC of 0.539. This indicates a considerable trade-off between sensitivity and specificity for clinician-collected samples in detecting CIN2, as shown in Figure 4a and Table 2. For CIN3 detection, self-collected samples with a Ct cut-off of 27.16 or less showed a sensitivity of 66.67% (95% CI: 9.4–99.2) and a specificity of 67.50% (95% CI: 56.1–77.6), with an AUC of 0.533. In contrast, clinician-collected samples with a Ct cut-off of greater than 22.31 achieved a sensitivity of 100% (95% CI: 81.5–100.0); however, the specificity dramatically decreased to 21.24% (95% CI: 14.1–29.9), and the AUC was 0.566, as shown in Figure 4b and Table 2. While clinician-collected samples for CIN3 demonstrated perfect sensitivity, it was at the expense of a very low specificity, indicating a high false-positive rate.

The AUC values, which represent the test’s overall diagnostic ability to distinguish between disease states, were relatively low across all categories, suggesting that Ct values alone may not be a strong discriminator for CIN2 or CIN3 risk assessment. The results illustrate the inherent challenges in balancing sensitivity and specificity in the context of CIN2 and CIN3 risk evaluation, especially when considering the method of sample collection. The summary emphasizes the contrast between sensitivity and specificity, particularly highlighting the extremes of high sensitivity with low specificity in clinician-collected samples for CIN3, which may necessitate further investigation or combination with other diagnostic methods to enhance clinical decision-making.

## 4. Discussion

The global fight against cervical cancer, particularly in low- and middle-income countries (LMICs), is fraught with challenges, notably in enhancing the reach and effectiveness of screening programs. The advent of self-sampling for human papillomavirus (HPV) has been recognized as a transformative approach that could significantly improve access to screening and maintain diagnostic accuracy. This strategy has proven especially vital in settings constrained by the COVID-19 pandemic, underscoring its potential to maintain continuity in cervical cancer screening efforts when traditional healthcare access is disrupted [1].

### 4.1. Age-Related Screening Efficacy

Age-related differences in HPV infection rates necessitate a strategic approach to screening. Younger populations are more likely to experience transient HPV infections that rarely progress to serious conditions, thus requiring protocols that avoid unnecessary treatment interventions, which could lead to overdiagnosis [11]. Conversely, older populations are more susceptible to persistent HPV infections, which are more likely to develop into cervical cancer if not detected and treated early. The analysis from Chonburi Hospital highlights this dynamic, showing that self-collected samples tend to have higher Ct values compared to those collected by clinicians, particularly in the older age groups. This suggests potential issues in sample collection efficacy and underscores the critical need for rigorous training and standardized protocols in self-sampling to ensure consistent and reliable outcomes [8]. Optimization of self-sampling (SS) assays holds critical importance for enhancing the effectiveness of HPV screening programs. Studies have emphasized the suboptimal performance of cytology as a triage method for hrHPV(+) self-collected samples, highlighting its poor sensitivity and cost-effectiveness in this context [9,12,13]. Consequently, the adoption of more effective triage methods such as DNA methylation analysis is recommended, which can utilize the same vial as the initial HPV test, reducing the need for additional sample collection by healthcare providers. This shift aligns with findings from Teghavi et al. [14], which demonstrated the feasibility of using alternative molecular triage methods that offer better sensitivity and specificity in detecting precancerous changes in self-sampled materials. Integrating these more sophisticated diagnostic techniques could significantly refine the screening process, enhancing early detection and subsequent management of cervical cancer risk in women.

Moreover, the variation in engagement with self-sampling methods across different age demographics suggests a disparity in accessibility or acceptability. Factors such as reduced dexterity, lower technological literacy, or skepticism about the effectiveness of self-sampling methods may deter older individuals from participating. Addressing these barriers through targeted educational and support interventions could significantly enhance the uptake and effectiveness of self-sampling among these higher-risk groups [8].

### 4.2. Influence of hrHPV Type on Screening Outcomes

The risk associated with different types of hrHPV, particularly types 16 and 18, is well documented, with these strains posing a higher risk of progression to cervical cancer. The study from Chonburi Hospital reveals a significant disparity in Ct values between self-collected and clinician-collected samples, indicating that clinician-collected samples are more effective in capturing viral DNA, a crucial factor for reliable HPV detection and subsequent management of high-risk infections [2,10,14,15,16,17,18]. Although there is a high level of agreement in HPV detection across both types of samples, the variability in the sensitivity and specificity across different HPV types indicates the need for continual refinement of self-sampling methods. Enhancing the diagnostic accuracy of these methods is crucial to ensure they are on par with clinician-collected samples, particularly in settings where professional sample collection may not be feasible [4,9].

### 4.3. Comparative Analysis of HPV Detection and Cytological Outcomes

The comparative analysis conducted at Chonburi Hospital indicates that self-collected samples tend to exhibit higher rates of cytological abnormalities, such as LSIL and HSIL. This could potentially indicate a higher occurrence of false negatives in clinician-collected samples or variations in the effectiveness of sample collection methods. Such findings highlight the importance of improving training and advancing technology to ensure self-collected samples achieve diagnostic outcomes comparable to those obtained by clinicians [13,18,19,20,21]. This is particularly crucial, as the adequacy of sample collection directly impacts the reliability of screening results and subsequent clinical decisions [22,23,24,25,26,27,28,29].

### 4.4. Perspective, Attitude, and Knowledge about HPV among Thai Women

Cultural perceptions and varying levels of knowledge about HPV significantly influence attitudes toward screening and vaccination across Thailand. Surveys indicate that while general awareness of HPV is moderate among Thai women, in-depth understanding of HPV transmission, its link to cervical cancer, and the benefits of vaccination remains low [29]. This lack of detailed knowledge can significantly impact women’s participation in screening programs and their acceptance of vaccination. In rural areas, traditional beliefs and stigma associated with sexually transmitted infections can further inhibit women from participating in screening programs, while urban women, often more exposed to health education, demonstrate a greater willingness to engage in such health initiatives [28,29].

Addressing these educational gaps through culturally sensitive campaigns and programs is essential to enhance understanding and trust in HPV screening, particularly self-sampling. Such efforts must be meticulously designed to resonate with the diverse cultural landscapes of Thailand, ensuring that all women, regardless of their socio-economic or educational backgrounds, receive accurate and accessible information about HPV and cervical cancer prevention [1,29,30,31].

## 5. Conclusions

The analysis of Ct values and the detection of various hrHPV types at Chonburi Hospital underscores significant considerations for the implementation of HPV testing in cervical cancer screening. Optimizing self-sampling strategies to improve viral load detection, alongside comprehensive screening and vaccination programs addressing the diversity of hrHPV types, is pivotal in advancing cervical cancer prevention and control efforts. This strategic approach could significantly enhance public health outcomes by facilitating earlier detection and treatment of precancerous changes, ultimately improving cervical cancer survival rates.

## Figures and Tables

**Table 1:** Comparison of Ct values and cervical lesion severity in self-collected and clinician-collected HPV samples.

	Self-Collected		Clinician-Collected		Mean Difference	*p*-Value
	n	Mean ± SD	n	Mean ± SD	(95% CI)	
Total	1541	29.22 ± 4.54	1398	27.69 ± 4.96	1.53 (1.18–1.87)	*p* < 0.0001
Age (years)						
30–39	859 (55.75%)	29.15 ± 4.43	578 (41.35%)	27.48 ± 4.81	1.67 (1.18–2.15)	*p* < 0.0001
40–49	524 (34.00%)	29.09 ± 4.66	518 (37.05%)	27.71 ± 5.03	1.38 (0.79–1.96)	*p* < 0.0001
50–59	158 (10.25%)	30.00 ± 4.67	302 (21.60%)	28.06 ± 5.10	1.94 (0.99–2.90)	*p* < 0.0001
HPV-DNA						
16	345 (22.39%)	30.36 ± 4.47	275 (19.67%)	28.13 ± 5.25	2.23 (1.46–3.00)	*p* < 0.0001
18	131(8.50%)	31.58 ± 4.93	135 (9.66%)	29.93 ± 5.24	1.65 (0.41–2.88)	*p =* 0.0089
non 16,18	1065 (69.11%)	28.56 ± 4.33	988 (70.67%)	27.26 ± 4.75	1.30 (0.90–1.69)	*p* < 0.0001
Cytology	282		963			
Negative	183 (64.89%)	29.44 ± 3.78	767 (79.65%)	28.37 ± 4.17	1.07 (0.41–1.73)	*p =* 0.0016
Positive	99 (35.11%)		196 (20.35%)			
LSIL	86 (30.49%)	26.88 ± 4.56	170 (17.65%)	23.02 ± 4.66	3.86 (2.41–4.88)	*p* < 0.0001
HSIL	13 (4.62%)	26.80 ± 4.29	26 (2.70%)	22.39 ± 3.13	4.41 (1.96–6.85)	*p* < 0.0008
Histology	110		160			
≤CIN1	80 (72.72%)	29.04 ± 5.06	113 (70.64%)	27.22 ± 5.76	1.82 (0.24–3.40)	*p =* 0.0238
CIN1–3*	22 (20.00%)	32.32 ± 4.88	19 (11.86%)	27.45 ± 5.08	4.87 (1.71–8.01)	*p =* 0.0034
CIN2	5 (4.55%)	29.75 ± 5.06	10 (6.25%)	26.65 ± 4.61	3.1 (2.53–8.72)	*p =* 0.2553
CIN3	3 (2.73%)	29.02 ± 3.30	18 (11.25%)	28.90 ± 5.55	0.12 (6.87–7.12)	*p =* 0.9713

CIN1–3* refers to a classification used in colposcopy results where a specific diagnosis between CIN1 and CIN3 cannot be definitively determined.

**Table 2:** Diagnostic performance of HPV Ct values in cytology and colposcopy outcomes: a comparison of self-collected and clinician-collected samples.

	Ct (Cut-Off)	Sensitivity (95% CI)	Specificity (95% CI)	AUC
Cytology				
Self-LSIL	≤26.07	48.84 (37.9–59.9)	82.51 (76.2–87.7)	0.668
Clinician-LSIL	≤24.95	69.41(61.9–76.2)	77.84 (74.7–80.7)	0.793
Self-HSIL	≤24.46	46.15 (19.2–74.9)	86.63 (84.3–93.6)	0.680
Clinician-HSIL	≤25.47	88.46 (69.8–97.6)	73.01 (69.7–76.1)	0.866
Colposcopy				
Self-CIN2	>33.68	40 (5.3–85.3)	85.00 (75.3–92.0)	0.555
Clinician-CIN2	≤29.65	80 (44.4–97.5)	38.05 (29.1–47.7)	0.539
Self-CIN3	≤27.16	66.67 (9.4–99.2)	67.50 (56.1–77.6)	0.533
Clinician-CIN3	>22.31	100 (81.5–100.0)	21.24 (14.1–29.9)	0.566

## Data Availability

The data presented in this study are available on request from the corresponding author.
